# Subarachnoid Hemorrhage in Patients with SARS-CoV-2 Infection: Protocol for A Scoping Review

**DOI:** 10.3390/brainsci12101327

**Published:** 2022-09-30

**Authors:** Amalia Cornea, Mihaela Simu, Elena Cecilia Rosca

**Affiliations:** 1 Department of Neurology, Victor Babes University of Medicine and Pharmacy Timisoara, 300041 Timișoara, Romania; 2 Department of Neurology, Clinical Emergency County Hospital Timisoara, 300736 Timisoara, Romania

**Keywords:** subarachnoid hemorrhage, SARS-CoV-2, COVID-19, systematic review, protocol

## Abstract

Subarachnoid hemorrhage (SAH) is a life-threatening condition associated with high mortality and substantial long-term morbidity. The SARS-CoV-2 virus is a new pathogen that causes a disease with variable clinical manifestations. Although the Coronavirus disease 2019 (COVID-19) is associated with hypercoagulopathy, patients may also present with cerebral hemorrhage, including SAH. The present paper reports a protocol for a scoping review that is aimed to provide a comprehensive report on existing literature by examining data on SAH associated with SARS-CoV-2 infection. Our objective is to evaluate the epidemiology, clinical, laboratory, and neuroimaging features of SAH in patients with COVID-19 and to explore the etiology and possible interventions in this pathology. Using appropriate search terms, we will search LitCOVID, the WHO database on COVID-19, and MedRxiv. The inclusion criteria are pre-defined. We will extract the data of eligible studies in standardized forms and will report the results in accordance with the Preferred Reporting Items for Systematic reviews and Meta-Analyses extension for Scoping Reviews (PRISMA-ScR). We will provide information for clinicians, healthcare providers, and public health specialists.

## 1. Introduction

Subarachnoid hemorrhage (SAH) is a life-threatening condition resulting from the accumulation of blood between the arachnoid and the pia mater membranes. Acute bleeding into the subarachnoid space can be because of several causes. The most frequent bleeding cause is the nontraumatic spontaneous SAH. The majority of primary SAH is due to the rupture of an intracerebral aneurysm in adults or cerebral arteriovenous malformation in children. However, in adults, the patients with primary SAH may present with no evidence of cerebral aneurism or other vascular malformations (non-aneurysmal SAH) in about 10% of cases (perimesencephalic and other angiogram-negative SAH) [1,2,3,4]. 

The secondary SAH etiologies include trauma, the reversible cerebral vasoconstriction syndrome, posterior reversible encephalopathy syndrome (PRES), cerebral amyloid angiopathy, cerebral vasculitis, cerebral venous sinus thrombosis, coagulopathies, tumors, drugs, septic emboli from endocarditis, or iatrogenic causes [1,2]. 

Although SAH is a rare cause of stroke, accounting for 1–6% of all strokes [5], the patients present high mortality and substantial long-term morbidity [5,6]. Although in the past decades, the mortality rates of SAH have decreased, it remains a severe neurological problem. For example, it is estimated that 10–15% of patients die before reaching the hospital [7], and about 25% of patients die within 24 h [7,8]. In the first month, hospitalized patients have an average mortality rate of 40% [8,9,10], and approximately half of affected individuals die in the first six months. The morbidity and mortality increase with age and are dependent on the overall health status of the patient [5,6]. Among survivors, more than one-third will present major neurologic deficits. In addition, cognitive deficits were reported even in patients considered to have a good outcome [11].

The timely recognition and adequate treatment of SAH are essential, and key management recommendations include several aspects related to SAH complications [6,12]. Among complications, the most important include: hydrocephalus, rebleeding, delayed ischemia, intracerebral hemorrhage, intraventricular hemorrhage, increased intracranial pressure, seizures, left ventricular systolic dysfunction, and myocardial infarction [1,4,13]. 

The SARS-CoV-2 virus is a new pathogen that causes a disease with variable clinical manifestations. Although the Coronavirus disease 2019 (COVID-19) is associated with hypercoagulopathy, patients may also present with cerebral hemorrhage, including SAH, spontaneous intracerebral parenchymal hemorrhage, and diffuse petechial cerebral hemorrhage. The incidence of intracranial hemorrhage, including SAH, was reported to be 0.3–1.2% [14].

However, studies on the neurological complications of SARS-CoV-2 infection report different epidemiological data according to the COVID-19 waves. This could be due to the impact of different healthcare strategies or the virulence of the different SARS-CoV-2 strains. For example, one study reports that SAH was present in 2% of the neurological cases in the first and 4% in the second wave [15]. Interestingly, a study comparing 2086 patients with COVID-19 and ischemic stroke with a cohort of 166,586 ischemic stroke controls found that the COVID-19 patients were less likely to present hypertension, dyslipidemia, and smoking history. However, they were more likely to be male, younger, and present with diabetes, obesity, acute coronary syndrome, venous thromboembolism, acute renal failure, and comorbid intracerebral hemorrhage or SAH [16]. Compared to the years before the pandemic, the research found that there was a higher proportion of ischemic stroke and intracerebral hemorrhage but a lower percentage of SAH and transient ischemic attack among patients with SARS-CoV-2 infection [17]. In patients hospitalized with pneumonia due to COVID-19, among 1040 individuals, 79.42% presented neurological symptoms. However, cerebral hemorrhage occurred in 1.08% of patients and SAH in 0.24% [18]. In mechanically ventilated patients with COVID-19 who died, although the leading cause of death was hypoxemic respiratory failure (77.8%), cerebrovascular accident accounted for 3.2% of deaths, and SAH for 1.6% [19]. 

Several studies indicated a decrease in the incidences of patients with acute cerebrovascular conditions [20,21,22,23,24,25] and aneurysmal SAH [20,26,27] during the early stages of the COVID-19 pandemic. A more extensive cross-sectional study, including 49 countries and 187 centers, investigated the differences in the incidence, severity of aSAH, and the treatment modality of ruptured aneurysms during the first year of the COVID-19 pandemic comparing it with the preceding year [28]. The authors found that there were 16,247 aSAH admissions and 344,491 COVID-19 admissions, with 8300 ruptured aneurysms coiling and 4240 ruptured aneurysms clipping procedures. They report a decline in the number of aSAH admissions (−6.4%; 95%CI −7.0% to −5.8%; *p* = 0.0001) during 2020 compared with 2019, most pronounced in hospitals with high-volume SAH and high-volume COVID-19 cases. In addition, the authors noted a trend towards a decline in mild and moderate SAH presentations (mild: −5%; 95% CI −5.9% to –4.3%, *p* = 0.06; moderate: −8.3%; 95% CI −10.2% to –6.7%; *p* = 0.06). Nonetheless, there was no difference in higher SAH severity [28]. The study also noted a similar overall 4.1% decrease in non-traumatic SAH admission. Consequently, the authors endorse the possibility that the aSAH rates may not have been modified, but the patients were shifted to being treated at hospitals with lower-volume COVID-19 cases if high-volume hospitals were overwhelmed with patients with SARS-CoV-2 infections [28]. 

Hemorrhages may affect multiple organs, including the central nervous system (CNS) [29]. The pathophysiology is not yet fully elucidated, and multiple factors were implicated: microthrombosis with secondary hemorrhage, dysregulated coagulation, vascular hyperpermeability in the context of SARS-CoV-2 infection cytokine storm, endotheliitis, and vasculitis [29]. For example, the affinity of SARS-CoV-2 for angiotensin-converting enzyme 2 (ACE-2) receptors, which are expressed in endothelial and arterial smooth muscle cells in the brain, may trigger local inflammation that causes a vasculitic process. Therefore, the viral infection will damage the intracranial arteries, predisposing the vessel wall to rupture, a mechanism that could potentially explain the pathogenesis of hemorrhagic stroke [30]. 

In patients with COVID-19 and aneurysmal SAH, the rupture of the aneurysm was assumed to be produced due to endothelial dysfunction. In addition, as the SARS-CoV-2 downregulates the expression of the ACE-2 receptors, the disruption of the renin-angiotensin-aldosterone system may cause an uncontrolled elevation in blood pressure, which is exacerbated by preexisting hypertension; thereby, the risk of bleeding is substantially increased [31]. Some authors proposed that the hyperinflammatory state also seen in COVID-19, with hypercytokinemia and inflammation, could contribute to vascular degeneration that will lead to aneurysm formation and size or morphology change, consequently resulting in rupture and bleeding [32].

It is noteworthy that the hypothesis of an infectious cause for aneurysm rupture was rejected several decades ago. Earlier studies on the association between viral infections and SAH failed to support the hypotheses that intracranial aneurysms may develop because the initiating event of a viral infection produces any direct arterial damage, and aneurysm rupture may be temporally related to the infection [33]. The authors tested the following viruses: influenza A, influenza B, the respiratory syncytial virus (RSV), herpes simplex virus (HSV), and rubella [33]. 

Our objective is to evaluate the clinical, laboratory, and neuroimaging features of SAH in patients with COVID-19 and to explore the etiology and possible interventions in this pathology. We aim to provide a comprehensive report on existing literature by investigating data on SAH associated with SARS-CoV-2 infection. A scoping review will also provide an extensive perspective on SAH in patients with SARS-CoV-2 infection, revealing what other, more specific research questions can be further addressed [34]. In addition, we aim to emphasize research gaps requiring attention.

## 2. Materials and Methods

We will perform a scoping review as we consider it the most appropriate knowledge synthesis method that would enable us to answer our research questions [35]. We used an online tool designed to provide guidance to reviewers on methods for conducting and reporting knowledge synthesis (https://whatreviewisrightforyou.knowledgetranslation.net/) (accessed on 1 September 2022) [36].

The present review aligns with the current recommendation for scoping reviews [37,38,39,40,41]. We followed the methodological approach we used for a previous scoping review, adapted to the present research questions [34,42].

In order to identify the extent of the current research on SAH in patients with SARS-CoV-2 infection, we performed a scoping search. Our aim was to see if another systematic review had already been done, to assess whether there is enough literature available on this topic, and to develop the search terms we should use to retrieve information on our topic. We searched LitCOVID, the World Health Organization database on COVID-19 (to December 20, 2021), using the term "subarachnoid." We also searched the MedRxiv pre-print server with appropriate terms. We retrieved 468 results. After deduplication, 371 articles were included. Screening the abstracts further reduced the number of articles to 156. Finally, we identified 47 papers on SAH in patients with COVID-19, with case reports or case series. All the screening and selection of evidence in the scoping search were performed by one author and cross-checked by another reviewer. The disagreements were managed either by discussion between the two screeners or by including a third author to arbitrate.

No systematic review was published in the first two pandemic years with a specific focus on SAH. However, several systematic reviews addressed the stroke topic. For example, one review up to 23 July 2020, found that, among 275 patients with stroke and SARS-CoV-2 infection, acute intracranial bleeding was identified in 35 cases: 24 patients (68.57%) presented with intracerebral hemorrhage (ICH), four patients (11.43%) had non-traumatic SAH, and seven individuals (20%) had the simultaneously SAH and ICH [43]. 

Among the 47 articles identified in our scoping search, several authors report on aneurysmal SAH (aSAH) in the context of COVID-19 [31,44,45,46] and non-aneurysmal SAH. The latter type of SAH was found eighter in isolation or in the context of other associated pathologies like reversible cerebral vasoconstriction syndrome [47,48,49], cerebral venous thrombosis [50], or intraparenchymal hemorrhage [30,51]. 

Therefore, a scoping review on SAH associated with SARS-CoV-2 infection is timely, as it can provide valuable insights into the spectrum of this type of stroke.

### 2.1. Research Questions

To identify fundamental problems to be addressed, we considered several aspects of SAH in COVID-19 patients. The research questions were defined based on the Population, Concept, and Context (PCC) of the review, as recommended by the Joanna Briggs Institute [37]:

- Is there a relationship between SARS-CoV-2 infections and the apparition of SAH?
- If yes, what types of SAH are present in COVID-19 patients?
- Which are the presumptive mechanisms underlying SAH?
- What are the clinical features?
- What do we know about laboratory and neuroimaging investigations?
- What interventions might work?

### 2.2. Search Strategy and Eligible Studies

Based on our PCC mnemonic [37], we will use a broad search strategy in the following databases: LitCOVID and WHO COVID-19 database. These databases are curated for SARS-CoV-2 infection articles; therefore, we will not need to use search terms like "coronavirus" OR "COVID-19" OR "SARS-CoV-2". We will use only the keyword: "subarachnoid." LitCOVID provides access to 269627 (and growing) relevant articles in PubMed. The WHO database indexes articles from Embase, Web of Science, Scopus, ProQuest Central, grey literature, and several other databases. In addition, we will search a pre-print server (MedRxiv) using appropriate terms. MedRxiv is one of the largest repositories in medicine; currently, it has approximately 35.520 manuscripts in the health sciences domain, with 18.958 COVID-19/SARS-CoV-2 pre-prints. Search filters will not be used, as we aim to generate a comprehensive list of research. However, we will limit our included papers to English literature. Recent research indicates that excluding non-English language records from a systematic review presented only a minimal effect on results [52]. In addition, the references of the included articles will be hand-searched to identify any additional research. We will use bibliographic software (EndNote 20, Clarivate Analytics, Philadelphia, PA, USA) to store, organize, and manage all retrieved references.

### 2.3. Study Selection

The PCC mnemonics for the present scoping review will be: adults (over 18 years old) and children (P), with studies investigating patients with SAH (C) in the context of SARS-CoV-2 infection (C). We will include prospective or retrospective observational or interventional studies. If available, apart from primary studies, we will also include systematic reviews. There will be no restrictions on age, gender, or region. 

We will include conference abstracts only if the authors did not publish a full article on the study. Conference abstracts are excluded from systematic reviews as they may not contain sufficient information for quality assessment or meta-analysis. However, we will include abstracts as they are frequently published earlier than the full articles [53], the time being essential to a rigorous review of an ongoing phenomenon.

Commentaries, correspondences, editorials, and opinion articles will be excluded. We will exclude narrative reviews, but we will assess their reference lists for possible inclusions. The inclusion and exclusion criteria are outlined in Table 1.

After the search, we will collate and upload all the identified citations into EndNote 20 (Clarivate Analytics, Philadelphia, PA, USA) and will remove all the duplicates. For the screening process, two authors will independently review the title and abstract of all identified reports, assessing the eligibility based on the criteria for inclusion and exclusion. All articles that are considered eligible by one or both reviewers will be assessed in full text. In the full-text stage, two authors will independently check, in detail, the selected articles against the inclusion criteria; in case of disagreements, a third reviewer will arbitrate. We will record and report in the final scoping review the reasons for the exclusion of papers that do not meet the inclusion criteria. 

We will provide a Preferred Reporting Items for Systematic reviews and Meta-Analyses extension for Scoping Re-views (PRISMA-ScR) flow diagram with the details of the search results and included and excluded studies, presenting each stage of the study selection process [38].

### 2.4. Data Extraction

The results will be presented in a tabular form in order to present a descriptive summary of the finding. The extracted data will comprise specific details about the participants, the concept, and the context of the study. In addition, we will collect the following essential information: the source of the data (e.g., author, year of publication, where the study was conducted), the research methods, and findings relevant to our review questions. 

The following patient data will be reported: age, gender, immunological status (vaccination, previous COVID-19 infection or immunosuppression), comorbidities and medication, clinical signs and symptoms (general and neurological), the time between the SARS-CoV-2 infection and SAH, laboratory findings (including general and specific to SARS-CoV-2 infection findings, CSF analysis), imaging (including pulmonary CT/MRI, neuroimaging and other imaging studies if performed ), postmortem investigations, type and severity of SAH, COVID-19 severity, treatment (general, and specific for COVID-19, and neurological/neurosurgical), evolution, and the assumed mechanism of SAH (See Table 2).

We will group the information into two categories for intervention studies: specific neurological or neurosurgical treatment and other interventions. We will extract data on the comparator (in available) and other details like the duration of the treatment and relevant outcomes. 

In order to ensure that all relevant data is retrieved, we will produce piloted extraction forms for the first five studies. In addition, the data extraction table might be modified and revised as necessary during the data extraction stage. Therefore, we may further refine the tables. However, any modifications will be detailed in the final scoping review. 

Data will be extracted independently by two reviewers; a third reviewer will solve any discrepancies. Although a formal risk of bias assessment is not planned for this scoping review, we will note the pre-prints, which have not been formally peer-reviewed.

### 2.5. Reporting the Results and Summarizing the Findings

The results will be presented following the PRISMA recommendations for scoping reviews [38]. We will provide a narrative summary that will accompany the tabulated or charted results. We will provide a description of how the results relate to our review’s objectives and questions. A numerical summary of the study characteristics will be presented. In addition, we will classify the findings under the main conceptual categories (e.g., clinical characteristics, paraclinical data, and interventions). We will use figures and tables to outline the results in an organized manner, in line with our objectives. We will document in the final published scoping review any amendments to this protocol, with reference to saved searches and analysis, in order to ensure a transparent process of the conduct of the review.

Finally, toward the end of the review, we will elaborate on the findings to help identify gaps in this research area. 

### 2.6. Quality Assessment

Our main aim is to map the evidence that has been reported in the area of SAH in the context of COVID-19 disease, providing an overview of the existing literature, regardless of the risk of bias in the included studies [13]. Hence, we will not make a formal evaluation of the methodological quality of the included studies. This can be further assessed in future systematic reviews with more precise questions.

## 3. Discussion

Similar to other evidence synthesis methodological recommendations, the development of a protocol is a prerequisite prior to conducting a scoping review [39]. There is a growing recognition of the value of the protocols of scoping reviews, as they are an essential element that increases the value and reduces waste in research [54,55]. A prespecified protocol is important as it provides a roadmap on the direction and methodological aspects of the future review. A protocol may be seen as a recipe for a systematic review that pre-establishes the ingredients (inclusion criteria), where and how to find them (the databases to be searched and the strategy to be used), and methods. It supports the reviewers in avoiding bias as it reduces the risk of any arbitrary decisions and undesired duplication of other systematic reviews. It also fosters collaboration and enables the detection of selective reporting. In addition, it may allow the assessment and possibly feedback from the research community [39].

In the case of SAH in the context of SARS-CoV-2 infection, the most appropriate approach is a scoping review that allows for comprehensive research questions. Therefore, a descriptive presentation of the literature on this subject is the most suitable evidence-based method. It will enable us to formulate conclusions and straightforward answers to our questions and identify any research gaps in this area. The present review will provide details on key implications for future research and further need for primary studies or systematic reviews [37,56].

We will also present data on important findings that may be used to inform clinical practice. However, the implications for practice may be restricted by the fact that we will not provide a methodological quality appraisal of the included research, and the practice recommendations will not be graded [37]. 

We plan to discuss the results in the context of current knowledge and practice. In addition, we will also present any potential limitations of our scoping review. For example, a limitation of scoping reviews is the lack of quality appraisal. If most of the studies will include case reports with inherent biases, then outcomes like the frequency of clinical manifestations may be influenced by selective reporting of more uncommon presentations. However, case reports provide a cornerstone for learning by pattern recognition and the advancement of medical knowledge [57]. For example, a systematic review that included 172 cases of a rare disorder (glycogenic hepatopathy) enabled the characterization of patterns of liver enzymes and hepatic injury in the disease [58]. Another systematic review, including the lipodystrophy cases from the literature, warranted the proposal of core and supportive clinical features of the disease and a narrative presentation of the data on available treatment options [59]. Furthermore, case reports contain much more information on individual patients than cohort studies [60].

The strength of the scoping review will be the thorough, systematic presentation of all published literature on patients with SARS-CoV-2 infection and SAH. The results will provide valuable information for clinicians and researchers, as well as priorities for future research. Our protocol can serve as a model for other systematic reviews on neurological presentations of COVID-19 or as a model for other protocols/systematic reviews on different clinical aspects of a disease.

A possible limitation of our scoping is that we will not assess the risk of bias in the included studies. We also do not plan to perform data synthesis like a statistical meta-analysis. Meta-analyses are recommended only if the authors assessed the methodological quality or the heterogeneity of the included studies. Usually, in scoping reviews, the analysis of data is descriptive, with basic frequency analysis and percentages [39]. 

Ultimately, we will provide an overall conclusion based on the results, in line with our objectives and questions. 

## 4. Conclusions

To date, several reviews on cerebrovascular events in COVID-19 patients have been published. Nonetheless, to the best of our knowledge, none of the reviews reports such a detailed presentation of SAH, covering all the critical aspects of the disease. Hence, our research will be of interest to clinicians and other healthcare providers, and public health researchers. The present work can be used as a precursor to further systematic review or meta-analysis with more circumscribed questions and may assist the development of inclusion criteria and research questions.

In addition, the publication of the present protocol enables our future scoping review of a detailed, transparent methodology. Our protocol can serve as a model for other systematic reviews on neurological presentations of COVID-19 or as a model for other protocols/systematic reviews on different clinical aspects of a disease.

## Figures and Tables

**Table 1:** Inclusion and exclusion criteria.

	Inclusion Criteria	Exclusion Criteria
Population	Adults and children	N/A
Concept	Subarachnoid hemorrhage (SAH)	Patients without SAH Traumatic SAH
Context	SARS-CoV-2 infection	No SARS-CoV-2 infection
Study type	Case report, case series, cohort studies	N/A
	Prospective, retrospective	N/A
	Observational, interventional	Predictive modeling
	Systematic review	Narrative review
	Original research	Commentaries, correspondences, editorials, opinion articles
Language	English	Non-English
Year	From 2019	Before 2019

**Table 2:** Data extraction form.

Dimensions	Details
General information	Authors, year, country
Study characteristics	Type of study
Study participants	Number
	Age
	Gender
	Immunological status (vaccination, previous COVID-19 infection, or immunosuppression)
	Comorbidities and medication
	General clinical signs and symptoms
	Neurological signs and symptoms
	Time between SARS-CoV-2 infection and SAH
Laboratory findings	SARS-CoV-2 diagnosis
	General laboratory findings
	CSF analysis
Imaging data	Pulmonary imaging
	Neuroimaging
	Other imaging
Postmortem data	Postmortem examination
Type of SAH	Aneurysmal SAH/non-aneurysmal SAH
SAH severity	Hunt and Hess grade, World Federation of Neurosurgical Societies, Fisher grade, etc.
COVID-19 severity	Mild, medium, or severe
Intervention	Specific neurological/neurosurgical treatment
	COVID-19 treatment
	Other treatments/general treatment
Evolution	Outcome
	Modified Rankin Scale
Assumed mechanism of SAH	
Additional comments/notes	

## Data Availability

Not applicable.
